# Treatment of recurrent acute tonsillitis—a systematic review and clinical practice recommendations

**DOI:** 10.3389/fsurg.2023.1221932

**Published:** 2023-10-10

**Authors:** Orlando Guntinas-Lichius, Katharina Geißler, Antti A. Mäkitie, Ohad Ronen, Patrick J. Bradley, Alessandra Rinaldo, Robert P. Takes, Alfio Ferlito

**Affiliations:** ^1^ Department of Otorhinolaryngology, Jena University Hospital, Jena, Germany; ^2^ Department of Otorhinolaryngology-Head and Neck Surgery, Research Program in Systems Oncology, University of Helsinki and Helsinki University Hospital, Helsinki, Finland; ^3^ Department of Otolaryngology, Head and Neck Surgery, Galilee Medical Center, Affiliated with Azrieli Faculty of Medicine, Bar-Ilan University, Safed, Israel; ^4^ Department Otorhinolaryngology, Head and Neck Surgery, Nottingham University Hospitals, Queens Medical Centre Campus, Nottingham, United Kingdom; ^5^ ENT Unit, Policlinico Città di Udine, Udine, Italy; ^6^ Department of Otolaryngology, Head and Neck Surgery, Radboud University Medical Center, Nijmegen, Netherlands; ^7^ International Head and Neck Scientific Group, Padua, Italy

**Keywords:** tonsillitis, sore throat, pharyngitis, quality of life, outcome, treatment, tonsillectomy, tonsillotomy

## Abstract

**Background:**

There is an ongoing debate on the indications for tonsil surgery in both children and adults with recurrent acute tonsillitis. The aim is to provide practical recommendations for diagnostics and treatment for recurrent acute tonsillitis including evidence-based decision making for tonsillectomy.

**Methods:**

A systematic literature search in PubMed, Embase, Web of Science, and ScienceDirect from 2014 until April 2023 resulted in 68 articles. These were the basis for the review and a comprehensive series of consensus statements on the most important diagnostics and indications for both non-surgical and surgical therapy. A consensus paper was circulated among the authors and members of the International Head and Neck Scientific Group until a final agreement was reached for all recommendations.

**Results:**

The differentiation between sore throat and tonsillitis patient episodes is mostly not feasible and hence is not relevant for diagnostic decision making. Diagnostics of a tonsillitis/sore throat episode should always include a classification with a scoring system (Centor, McIssac, FeverPAIN score) to estimate the probability of a bacterial tonsillitis, mainly due to group A *streptococcus* (GAS). In ambiguous cases, a point-of-care test GAS swab test is helpful. Consecutive counting of the tonsillitis/sore throat episodes is important. In addition, a specific quality of life score (Tonsillectomy Outcome Inventory 14 or Tonsil and Adenoid Health Status Instrument) should be used for each episode. Conservative treatment includes a combination of paracetamol and/or non-steroidal anti-inflammatory drugs. In case of high probability of bacterial tonsillitis, and only in such cases, especially in patients at risk, standard antibiotic treatment is initiated directly or by delayed prescription. Tonsillectomy is indicated and is highly effective if the patient has had ≥7 adequately treated episodes in the preceding year, ≥5 such episodes in each of the preceding 2 years, or ≥3 such episodes in each of the preceding 3 years. An essential part of surgery is standardized pain management because severe postoperative pain can be expected in most patients.

**Conclusion:**

It is necessary to follow a stringent treatment algorithm for an optimal and evidence-based treatment for patients with recurrent acute tonsillitis. This will help decrease worldwide treatment variability, antibiotic overuse, and avoid ineffective tonsillectomy.

## Introduction

Group A β-hemolytic *streptococcus* (GAS) is responsible for 5%–15% of sore throat visits in adults and 20%–30% in children (1). Pediatric streptococcal pharyngitis (or tonsillitis) in the US costs an estimated $224–$539 million per year, including indirect costs related to parental work losses (2). Accurate diagnosis and appropriate antimicrobial therapy for streptococcal pharyngitis/tonsillitis are important to prevent acute rheumatic fever and suppurative complications, improve symptoms, decrease contagiousness, reduce transmission, and minimize the adverse effects of inappropriate antimicrobial therapy. However, the signs and symptoms of GAS and non-streptococcal pharyngitis and/or tonsillitis overlap, making clinical diagnosis impossible. Antimicrobial therapy is of no proven benefit for acute pharyngitis/tonsillitis caused by organisms other than GAS. Physicians must exclude the diagnosis of GAS pharyngitis to prevent inappropriate administration of antimicrobials and the development of antimicrobial resistance among common pathogens (3). If a patient has repeated GAS pharyngitis/tonsillitis, the treatment decision becomes even more difficult.

The debate on the optimal treatment and surgical indications for recurrent acute tonsillitis is still ongoing. An important parameter for decision making is the number of bacterial tonsillitis episodes the patient had in the preceding year or years, taking into account the social, work, or educational absence or alteration to life each episode has had. Here the questions start: How best to count these episodes? How do we know which episodes were caused by a bacterial infection or not? How do we know that it was a tonsillitis and not just an acute laryngopharyngitis? Hence, the indication for or against surgery is based on non-reliable criteria. On the other hand, hard outcome criteria are also difficult to define. There is no objective measurement that directly correlates with the severity of tonsil inflammation. Most clinical trials that have defined the indication for tonsil surgery based on tonsillitis episodes have used cumulated sore throat episodes over time after surgery as outcome criterion (4–6). Here, the problem is the same: When does a sore throat episode start? All these issues together might explain why it has been so difficult in the past to prove with a high level of evidence whether tonsil surgery is effective in severe cases of recurrent acute tonsillitis (5). These (unclear) benefits of surgery have to be balanced against the risk of surgery. Worldwide, the surgical standard for the treatment of recurrent acute tonsillitis is tonsillectomy. Tonsillectomy has a relevant postoperative complication risk, including bleeding. Overall, the frequency of readmission for post-tonsillectomy hemorrhage is about 2%–7%, and the reoperation rate for hemostasis is about 1%–2% (7–9). Although rare, some patients even die due to bleeding after tonsillectomy (three to seven deaths per 100,000 tonsillectomies) (10, 11). Furthermore, tonsillectomy is one of the most painful procedures, even more painful than large abdominal surgeries (12, 13). This is why it is currently investigated if tonsillotomy, i.e., a procedure with much less morbidity and only minor pain, is as effective to treat recurrent acute tonsillitis in children and adults (14).

The review presented here gives an overview about the current knowledge on recurrent acute tonsillitis and provides recommendations for optimal diagnostics and treatment of children and adult patients with recurrent acute tonsillitis.

## Material and methods

### Search and consensus strategy

As a starting point, a careful review was done on the current clinical guidelines (Supplementary Table S1). This was followed by a systematic review conducted in three steps in accordance with the preferred reporting items for systematic reviews and meta-analyses (PRISMA) guideline (15). We conducted a systematic literature search for the publications in English language since 2014 in PubMed, Embase, Web of Science, and ScienceDirect databases. The year 2014 was chosen, because all relevant literature older than 2014 were evaluated in the German clinical guideline on tonsillitis treatment in children and adults (16). The following MeSH terms were used: “tonsillitis”, “Recurrence”, “Humans”, “Tonsillitis* / surgery”, “Tonsillitis* / therapy”, “Tonsillectomy* / adverse effects”, “Tonsillitis* / epidemiology”, “Quality of life”, “Child”, “Adult”, “Palatine tonsil”, “Sore throat”, and “humans” (period: 2008–2022; last search on May 5, 2022). Our research retrieved 314 records. In agreement with the PRISMA guidelines (15), we reported the results using the PICOST-DS tool (17): Participants: all ages, recurrent acute tonsillitis; intervention: any kind of intervention: drug therapy, non-surgical therapy, and surgical therapy; comparator: not needed; outcomes: no restriction; time: no limits of the time; setting: outpatients and inpatients; and study design: all designs studies. Finally, a total of 68 studies were included in the present review based on relevance and scientific evidence. A flow diagram of the research is reported in the Supplementary Material (Supplementary Figure S1).

### Recommendation assessment

The highest level of evidence reached the level of retrospective observational cohort studies (Oxford Centre for Evidence-based Medicine Level III–IV). Due to the lack of higher quality evidence, the presented recommendations reached the level of recommendation B, i.e., considerable benefit substantiated by non-first-class evidence, according to international standards and the Association of the Scientific Medical Societies guidelines (18). The most important diagnostic tests and treatment options were discussed in-depth and a consensus was proposed. Based on the evidence of the literature, a proposal for all recommendations was written by the first author. All authors made comments on each recommendation and were able to view the comments of all authors in the process. Based on the first round of comments, the recommendations were revised by the first authors. Again, the recommendations circulated among the authors to give another opportunity to comment. Based on the second round of comments, the recommendations were revised again by the first author. All authors finally agreed with the formulations. In total, the articles were circulated among all authors for three rounds until a consensus was reached for all recommendations. A strong consensus (100% of all eight authors) was reached for all recommendations based on the Delphi process.

## Results

### Definition of recurrent acute tonsillitis and tonsil surgery

“Recurrent acute tonsillitis” is the repeated distinct time period or episodes of acute bacterial infections of the palatine tonsils with symptom-free or symptom-poor intervals. It has to be emphasized that the term is restricted to the palatine tonsils and assumes a bacterial origin of the tonsillitis episodes. This has to be differentiated from the terms “sore throat” and “pharyngitis” which are frequently used interchangeably. Sore throat describes a painful irritation of the throat independent of an obvious etiology. In real life and for decision making, a differentiation between an episode of sore throat and an episode of tonsillitis is often not possible. Tonsillectomy is defined as complete removal of the palatine tonsils, including its capsule, by dissecting the peritonsillar space between the tonsil capsule and the muscular wall (19). In case of intracapsular or partial tonsillectomy, all tonsil tissue is removed, but a small remnant layer is retained (the “capsule”) to protect the underlying muscles. The term tonsillotomy is sometimes used synonymously to report the use of partial tonsillectomy. Alternatively, tonsillotomy is used to describe cutting through the tonsil at the level of the palatal arches, i.e., all tonsil tissue beyond this level is left in place.

Recommendation: A clarification of the terminology is needed

The correct term for repeated occurrence of acute bacterial tonsillitis is “**Recurrent Acute Tonsillitis**”. The obsolete term chronic tonsillitis is no longer in use. “**Tonsillectomy**” is the only clearly defined term. We recommend using the term “**Intracapsular Tonsillectomy**” if a small layer of tonsil tissue remains in place to protect the pharyngeal musculature. We recommend using the term “**Tonsillotomy**” if only the tonsil tissue that exceeds the level medial to the palatal arches is resected. We recommend not using the term partial tonsillectomy, because then the extent of tonsillar resection remains unclear.

### Pathophysiology of recurrent acute tonsillitis

The palatine tonsils are part of Waldeyer's ring and belong to the mucosa-associated lymphoid tissue (MALT) (20). A pathologist reporting on an excised normal palatine tonsil, i.e., a tonsil of a person who never had problems with this organ, would find many inflammatory cells, different sets of leukocytes including neutrophils, or granulocytes. The pathologist would thus report an inflammation of the tonsil. This inflammation of the tonsil reflects its physiological function. This inflammatory process only becomes a disease, when this physiological—local—inflammation is accompanied by a viral or bacterial infection with clinical symptoms. The predominant reason for a bacterial infection is symptomatic cases of GAS tonsillitis (21). Depending on the region, bacteria like *Streptococcus dysgalactiae subsp. equisimilis*, *Fusobacterium necrophorum*, and others can also be found in adolescents and young adults with tonsillitis symptoms (22). The patient has recurrent acute tonsillitis when she/he develops further episodes of acute bacterial tonsillitis with symptom-free or symptom-poor intervals after the first bacterial tonsillitis.

### Epidemiology of recurrent acute tonsillitis

Acute (and mainly viral) tonsillitis is a frequent disease, said to account for about one-third of all respiratory tract infections treated in primary care (23). Exact numbers are difficult to obtain, with all the previously mentioned difficulties of definition and unreliable diagnostic criteria. Despite these circumstances, it is estimated that 600 million symptomatic GAS cases are diagnosed worldwide each year (21). About one among 10 of these patients develop recurrent acute tonsillitis. Nevertheless, it affects hundreds of thousands of children and young adults every year. The prevalence of recurrent episodes is about 12% in patients with tonsillitis, i.e., about 12,000 per 100,000 individuals having had at least one tonsillitis episode before (24).

### Risk factors for recurrent acute tonsillitis

A GAS infection as a cause of a tonsillitis episode is the only known hard risk factor for the development of recurrent acute tonsillitis (25). Recurrent acute tonsillitis could be a genetic immunosusceptibility disease because it is reported that patients younger than 12 years with recurrent acute tonsillitis show some (otherwise subclinical) antibody deficiency and aberrant T-cell function (26).

### Clinical assessment of recurrent acute tonsillitis

Viral and bacterial infections have the same unspecific clinical symptoms like sore throat with or without fever. The clinical differentiation is often not possible. Furthermore, there is no examination that allows one to differentiate between a patient with acute tonsillitis, acute pharyngitis without tonsillitis (if this really exists), tonsillopharyngitis, or laryngopharyngitis. Therefore, many physicians prefer to ambiguously define it as “sore throat.” The number of bacterial tonsillitis episodes is currently the most important parameter to determine the indication for or against tonsillectomy. Therefore, the proof that the patient has another episode of bacterial tonsillitis is important. Empirically, about 70% of the cases are viral infections, and about 30% have a bacterial etiology (27). The probability in children is higher than in adults. As most of the bacterial infections are caused by GAS, the proof of a GAS-related tonsillitis is important.

The mostly used clinical scoring systems are only validated for GAS infections and patients with sore throat, i.e., not explicitly designed for patients with acute tonsillitis (Table 1). The scoring systems only allow a gross estimation of the probability of a bacterial tonsillitis. If the patients have a high score (Centor score 3–4 points; McIsaac score: 3–4 points; or FeverPAIN score: 3–5 points), the standard approach then, especially in high-risk patients, is to perform a fast point-of-care test (POCT) swab test. Modern POCTs have a high sensitivity and specificity comparable with time-consuming standard laboratory testing (28, 29). Nevertheless, one should be aware that children can be asymptomatic GAS carriers (30). Physicians who wish to ensure maximal sensitivity in diagnosis when the patient has a negative POCT test but relevant symptoms or even worsening symptoms may repeat the POCT test or continue to use conventional throat culture with standard laboratory testing (3). In any case, if the initial scoring indicated an uncomplicated tonsillitis and the patient's condition worsens, re-evaluation should be considered after 3 days.

**Table 1:** Most important scores for prediction of a *Streptococcus* infection in patients with sore throat.

Parameter	Centor score (99)	McIsaac score (100)	FeverPAIN (101)
Validated for	GAS	GAS	β-hemolytic *Streptococcus* (BHS) of groups A, C, and G
Target group, age	For patients aged ≥15 years	Primarily or patients aged 3–14 years	
Within 3 days after onset			1 point
Body temperature >38°C	1 point	1 point	1 point
No cough	1 point	1 point	1 point
Cervical lymph node swelling	1 point	1 point	
Tonsillar swelling/exudate	1 point	1 point	1 point
Tonsillar redness/inflammation			1 point
Age		<15 years: 1 point	
		≥45 years: minus 1 point	
Sum of points = score and probability	0: 2,5%	0: 2.5%	0: 14%
	1: 6–7%	1: 4.4%–5.7%	1: 16%
	2: 15%	2: 11%	2: 33%
	3:30–35%	3: 28%	3: 43%
	4: 50–60%	4–5: 38%–63%	4–5: 63%

A newer optional but practical approach is to begin with symptomatic treatment, for instance, with adequate dose of rapid-acting paracetamol directly in the office/clinic (22). If this does not have an effect within 15–30 min, a fast POCT swab test should be used.

Recommendation: Use a standard algorithm to evaluate the probability of a bacterial tonsillitis

1. Patient history and otorhinolaryngological examination.
2. Scoring with an adequate scoring system (Centor, McIssac, FeverPAIN score).
3. In case of high score, especially in high-risk patients: obtain POCT GAS swab test.

### Classification of the severity of recurrent acute tonsillitis

It was unclear until recently if a tonsillectomy is effective in patients suffering from severe recurrent acute tonsillitis. The Cochrane review from 2014 based on seven trials (five in children and two in adults; Table 2) revealed that good information about the effectiveness of tonsillectomy (with/without adenoidectomy) was only available for the first year following surgery in children and for 5–6 months in adults (6). Children had a small benefit from surgery: children who had surgery had three episodes of sore throat on average compared to 3.6 episodes experienced by the other children, i.e., 0.6 episodes of any type of sore throat in the first year was avoided. In adults, conclusions on the effectivity could not be drawn: the two included trials suggested that the number of days with sore throat may be fewer in the first 6 months following tonsillectomy compared to a conservative management. This was the reason why the UK's National Institute for Health Research commissioned the NATTINA trial (6). NATTINA is so far the largest multicenter clinical trial (453 participants randomized) to assess the effectiveness of surgical intervention in adults compared to a conservative management (Table 2). NATTINA represents a new milestone on the importance of tonsillectomy in adults (31). The primary outcome measure was the total number of sore throat days, reported weekly over 24 months (32). It was shown that compared with conventional medical management, tonsillectomy is both clinically effective and cost-effective. Tonsillectomy reduces sore throat days by almost 53% in over 2 years.

**Table 2:** Most important randomized controlled clinical trials analyzing the efficacy of tonsil surgery compared to a control group.

Authors	Age, years	Numbers included/screened	Eligibility	Intervention (I) Comparator (C)	Primary outcome	Quality of life measurement	Results
Paradise et al. (4)	3–15	91/2,034	• ≥7 episodes in the preceding year OR • ≥5 episodes in each of the 2 preceding years OR • ≥3 episodes in each of the preceding 3 years	I: Tonsillectomy or adenotonsillectomy C: Medical treatment	Episodes of throat infections over 3 years		I: 4.62 infections C: 7.95 infections Tonsillectomy had better outcome
Paradise et al. (102)^a^	3–15	177/2,174	Aged 3–6 years: • 4–6 qualifying units in the preceding year and (≥1 “counting” unit = plus other defined symptoms) OR • 4 units in each of the past 2 years (≥2 “counting” unit per year = plus other defined symptoms) Aged 7–15 years: • 4–6 qualifying units in the preceding year and (≥1 “counting” unit = plus other defined symptoms) OR • 3 units in each of the past 2 years (≥1 “counting” unit per year = plus other defined symptoms) • In addition, if criteria were not documented, for all children, aged 7–15 years: • No Paradise criteria documentation: documentation of ≥1 unit in last 4 months, OR • In case of incomplete documentation: ≥1 unit in last 3 months.	I1: Tonsillectomy I2: Adenotonsillectomy C: Medical treatment	Episodes of throat infections over 3 years		I1: 1.55 infections I2: 1.63 infections C: 2.77 infections Tonsillectomy had better outcome, but illness rates were moderate. Adenotonsillectomy was no more efficacious than tonsillectomy alone
Paradise et al. (102)^a^	3–15	151/2,174	• Same as Paradise et al., see above	I: Adenotonsillectomy C: Medical treatment	Episodes of throat infections over 3 years		I: 1.74 infections C: 2.93 infections Adenotonsillectomy had better outcome, but illness rates were moderate.
van Staaij et al. (103)	2–8	300/1,226	• Adenotonsillectomy indicated according to “current medical practice” in Netherlands. ENT surgeons were asked to provide the indication they considered most important for surgery: recurrent throat infections (3 or more a year) or other indications such as obstructive problems or recurrent upper respiratory tract infections	I: Adenotonsillectomy C: Waiting	Incidence of fever (a temperature of 38.0°C or higher) for at least 1 day for 2 years		I: 5.31 fever days; 0.56 infections C: 5.93 fever days; 0.77 infections Tonsillectomy had better outcome
Alho et al. (104)	≥15	70/298	• ≥3 episodes in 6 months OR • ≥4 episodes in 12 months	I: Tonsillectomy C: Waiting	Proportion of patients with GAS episodes within 90 days	Glasgow Benefit Inventory	I: 1 patient with recurrent infection C: 8 patients with recurrent infection Tonsillectomy had better outcome
Lock et al. (105)	4–15	268/1,546	• ≥4 episodes of sore throat within each of the preceding 2 years OR • ≥6 episodes of sore throat within the last year	I: Tonsillectomy C: Medical treatment	Monthly numbers of episodes of sore throat in the 2 years	PedsQoL, parent-reported	I: 0.50 first year, 0.13 s year C: 0.64 first year, 0.33 s year Tonsillectomy had better outcome
Koskenkorva et al. (106)	≥13	86/260	• ≥3 episodes in 12 months	I: Tonsillectomy C: Waiting	Proportion of patients with severe episode of pharyngitis within 5 months		I: 38% C: 41% Tonsillectomy had better outcome.
Wilson et al. (32)	≥16	453/4,165	UK guideline for tonsillectomy: • ≥7 sore throats in the preceding year, OR • ≥5 episodes in each of the preceding two years, OR • ≥3 episodes in each of the preceding three years	I: Tonsillectomy C: Medical treatment	Number of sore throat days over 2 years	Tonsil Outcome Inventory 14	I: Median 23 days C: Median 30 days Tonsillectomy had better outcome.

^a^ The results of two trials were presented in one publication.

Based on the main NATTINA result that tonsillectomy is more beneficial mainly for severe cases of recurrent acute tonsillitis, current clinical guidelines recommend tonsillectomy if the patient experiences at least five to seven sore throat episodes within 1–2 years (Supplementary Table S2). For example, the German guideline explicitly requires episodes of acute tonsillitis. In addition to the number of episodes, nearly all guidelines require additional parameters to substantiate the severity and a high probability that the episodes were episodes of bacterial tonsillitis. The NATTINA trial probably will define the new standard to count the number of episodes, i.e., following the UK guideline (cf. Table 2 and Supplementary Table S2).

Recommendation: To classify the severity of the recurrent acute tonsillitis in the individual patient

1. Count the number of episodes of sore throat for each of the last three years that were disabling and prevented normal functioning (i.e., going to work, school).
2. These episodes should have been treated with antibiotics.
3. There could be an indication to recommend tonsillectomy, if the patient had ≥7 adequately treated sore throats/tonsillitis episodes in the preceding year, ≥5 such episodes in each of the preceding two years, or ≥3 such episodes in each of the preceding 3 years.

To allow a better estimation of the disease burden, a quality of life assessment should be performed. The most frequently used generic questionnaire is the Glasgow Benefit Inventory (GBI) (33). It is used to report change in quality of life after surgery. The PedsQL is another generic tool frequently especially used for children (34). Specific questionnaires are the Tonsillectomy Outcome Inventory 14 (TOI-14) and the Tonsil and Adenoid Health Status Instrument (TAHSI) (35, 36). Both the TOI-14 and the TAHSI were designed to assess the disease-specific quality of life for patients with recurrent or chronic tonsillitis. Both questionnaires are partly validated in several languages for adults and children (English, Spanish, German, Arabic, Finnish) (37–40). The benefit of tonsillectomy in gaining quality of life is higher in patients with higher number of tonsillitis episodes before surgery (41, 42).

**Recommendation:** To assess the severity of the disease, patients’ quality of life should be measured using TOI-14 or TAHSI. The instruments can also be used to measure the therapy effect on quality of life.

### Therapy of recurrent acute tonsillitis

There are three treatment options: watchful waiting, drug therapy, or tonsillectomy. A decision is made based on the evaluation of the severity of the recurrent acute tonsillitis. If the patient has an apparently uncomplicated episode, is immunocompetent, not comorbid, and has a low score (Centor, McIsaac, FeverPAIN, see above), waiting or symptomatic treatment is indicated. In comorbid or critically ill patients, one should lean toward recommending POCT for GAS (22). Identification of patients with risk for severe disease course is important. Conditions are severe immunosuppression, such as long-term use of systemic steroids, organ transplantation, stem cell transplantation, AIDS, neutropenia, and other congenital or acquired immune defects, or severe comorbidities (43).

Peritonsillar abscess, or quinsy, is a relevant complication of acute bacterial tonsillitis. The annual incidence rates are in the range of 9–41 cases in a population of 100,000 (44). Whether the risk for a peritonsillar abscess is higher in patients with recurrent acute tonsillitis is unclear. Some studies describe higher risks, and others report that patients with peritonsillar abscess have less likely a history of recurrent acute tonsillitis (44–46). This means that the risk of peritonsillar abscess cannot be considered a standalone argument for or against a particular therapy for recurrent acute tonsillitis.

If POCT is unavailable, antibiotic treatment is recommended in these subgroups of patients. In patients with high score and poor effect on fast-acting symptomatic analgesics (see above), one would consider antibiotics in the presence of GAS due to POCT. Finally, in case of severe recurrent tonsillitis based on the number of tonsillitis episodes, tonsil surgery should be recommended.

### Conservative treatment

Standards for symptomatic treatment are non-steroidal anti-inflammatory drugs and paracetamol (43). If the situation worsens after 2 days or if there is no noticeable improvement over 8 days, the patient should come back for re-evaluation (22). Due to the limited effect of oral or intramuscular corticosteroids in reducing the pain, we do not recommend the use of corticosteroids (47). The US Food and Drug Administration (FDA) issued a black box warning for codeine and tramadol in children younger than 12 years and limited use in children between 12 and 18 years of age owing to difficulty breathing and death (48).

In case of a high score (Centor, McIsaac, FeverPAIN; see Table 1 and above) and a positive GAS POCT when required (see above), antibiotic treatment can be considered. The patient or the caregivers should know that the primary goal of antibiotic treatment is to shorten the duration of the disease rather than to prevent complications (43, 49). Several studies show that antibiotics reduce acute symptoms more frequently in patients if GAS was present (22). The patient or the caregivers should also know that these benefits have to be balanced against drug reactions like diarrhea, anaphylaxis, and mycoses occurring in up to 10% of the patients (43). In patients with an intermediate score, a delayed prescribing can be performed, i.e., issuing of the prescription is only redeemed by the patient if symptoms worsen or do not improve after 3–5 days (50). The recommended antibiotics, either by immediate administration or delayed prescribing, are listed in Table 3. Penicillin is the antibiotic of choice. There is no clear evidence that other antibiotics are more effective than penicillin in the treatment of an episode of recurrent GAS-related tonsillitis (51–53).

**Table 3:** Examples for antibiotic treatment of an acute tonsillitis episode probably related to a GAS infection.

Group of patients	First choice	In the case of penicillin intolerance or second choice
Adolescents (>15 years) and adults	Penicillin V (phenoxymethylpenicillin-potassium 0.8–1.0 million IU (or phenoxymethylpenicillin benzathine 100.000 IU/kg body weight) orally three times daily for 5–7 days	Clarithromycin 250–500 mg orally twice daily for 5 days OR erythromycin 40 mg/kg body weight divided into 2 single oral doses for 5 days OR clindamycin (20 mg/kg body weight/day divided into three single oral doses for 5 days)
Children (3–15 years)	Penicillin V 0.05–0.1 million IU/kg body weight/day divided into three single oral doses for 5–7 days	Clarithromycin 15 mg/kg body weight/day divided into two single oral doses for 5 days.
Individual cases at increased risk for a severe course	Pathogen eradication with penicillin for 10 days

Recommendation: Standards of conservative treatment are

1. Standards for symptomatic treatment are non-steroidal anti-inflammatory drugs and/or paracetamol.
2. In case of high GAS scoring or patient with risk for complications: antibiotic treatment by immediate administration or delayed prescribing.
3. In most regions of the world, penicillin is the standard antibiotic drug (see Table 3).

### Surgical treatment

Surgery is indicated for severe cases of recurrent acute tonsillitis. The severity has to be classified as described above. Surgery is indicated based on a clear cut-off number of acute tonsillitis episodes (see above). Standard surgery is bilateral tonsillectomy. The recently published NATTINA trial has confirmed the efficacy and also the cost-effectiveness of tonsillectomy in adults with severe recurrent acute tonsillitis (32). Only the German guideline offers as alternative a tonsillotomy in case of tonsil with a size >Brodsky grade 1 (16, 54). We do not recommend performing tonsillotomy or any other kind of partial tonsillectomy outside from clinical trials. So far, the evidence that tonsillotomy is as effective as tonsillectomy for recurrent acute tonsillitis is unproven (55). In the treatment of sleep-related disorders, this is different for children. Here, partial tonsillectomy/tonsillotomy is recommended as an alternative to tonsillectomy in many countries. It is important to distinguish between the two indications, sleep-related disorders and recurrent acute tonsillitis.

**Recommendation:** If surgery is indicated, perform a bilateral tonsillectomy. Other types of tonsil surgeries, for instance, tonsillotomy for recurrent acute tonsillitis, should only be performed in clinical trials.

### Surgical techniques

The most frequent and traditional method of tonsillectomy is with metal surgical instruments (cold steel tonsillectomy; tonsillectomy by cold dissection or with a snare/guillotine). Due to the relevant risk of primary and secondary postoperative bleeding and because of moderate to severe pain for up to weeks after surgery, other techniques are being used for decades with the aim to reduce the risk of postoperative bleeding and severe pain (56). Alternative techniques are bipolar radiofrequency ablation (coblation), bipolar electrodissection (electrocauterization, electrocautery), harmonic scalpel, microdebrider-assisted partial tonsillectomy, diathermy, laser surgery, and cryosurgery. Tonsillectomy by cold dissection and also the alternative techniques are more or less used for total tonsillectomy as well as for partial tonsil surgery. The current evidence remains low regarding any of the alternative techniques having advantages over traditional tonsillectomy (56). Most trials do not differentiate clearly between the indications (recurrent acute tonsillitis vs. tonsillar hypertrophy) and the extent of tonsil surgery (56). Population-based data do not report an advantage for alternative techniques (7, 57, 58). It is important to separate the question of the best technique from the extent of surgery. There is a consensus that any partial type of tonsil surgery, especially tonsillotomy, has a lower postoperative bleeding rate and produces less postoperative pain (8, 59, 60). Hence, any technique used for partial tonsillectomy and tonsillotomy has a lower risk of postoperative bleeding and severe pain.

**Recommendation:** No surgical technique is better than the other. Use the technology for tonsil surgery that is established in your office/hospital and with which the surgeons feels comfortable.

### Postoperative pain management

Tonsillectomy is ranked among the top 25 procedures with highest pain intensities (12, 61). It is more painful than a number of so-called major abdominal surgeries. Median pain scores are about 5–6 on a 11-part numeric rating scale (NRS 0–10). Typically, a planned pain management is recommended if an NRS >3 can be expected. Nevertheless, there is no consensus for optimal pain management. Moreover, some clinicians still are underestimating the degree of pain associated with tonsillectomy, as they consider it be a minimally invasive surgical procedure. Patients with preoperative chronic pain due to other diseases, females, and young adults are associated with higher postoperative pain intensity (13).

A recent systematic review analyzed preoperative and intraoperative interventions to reduce postoperative pain. Paracetamol (acetaminophen), non-steroidal anti-inflammatory drugs (NSAIDs), intravenous dexamethasone, ketamine (only assessed in children), gabapentinoids, dexmedetomidine, honey, and acupuncture improved postoperative pain. Inconsistent evidence was found for local anesthetic infiltration, antibiotics, and magnesium sulfate. Limited evidence was found for clonidine (62). Mouthwash gargle with benzydamine hydrochloride was not found to be superior to placebo in a recent randomized control trial (63). Evidence-based recommendations for pain management are listed in Table 4. Pain management should be initiated during tonsillectomy. Non-opioid analgesics should be administered intraoperatively. The administration should be continued in the postoperative period, unless contraindications are present (64). Intravenous dexamethasone should be given intraoperatively. The patient can have high pain scores typically for 3–5 days after surgery.

**Table 4:** Recommended pain management in patients undergoing tonsillectomy^a^.

Preoperative and intraoperative	Grade^b^
Paracetamol	D
Non-steroidal anti-inflammatory drugs	A
Dexamethasone intravenously	A
Preoperative gabapentinoids, or intraoperative ketamine (for children), or intraoperative dexmedetomidine may be considered, when basic analgesic regimen is contraindicated	D
Analgesic adjuncts	* *
Acupuncture	B
Postoperative	
Paracetamol	D
Non-steroidal anti-inflammatory drugs	A
Opioid for rescue	D
Analgesic adjuncts	* *
Acupuncture	B
Honey	B

A, consistent level 1 studies; B, consistent level 2 or 3 studies or extrapolations from level 1 studies; C, level 4 studies or extrapolations from level 2 or 3 studies; D, level 5 evidence or troubling inconsistent or inconclusive studies of any level.

^a^ In accordance with Aldamluji et al. (62) and Guyatt (107).

^b^ Grades (https://www.gradeworkinggroup.org/).

In general, mono-analgesics only have a limited analgesic efficacy in the postoperative setting after tonsillectomy. Therefore, it is recommended to use analgesic in combination (e.g., paracetamol plus NSAIDs). Ibuprofen is widely used as a first choice NSAID (65). There is no evidence that NSAIDs increase the risk of postoperative bleeding (66, 67). The value of intravenous dexamethasone in the postoperative phase is controversial and it is not frequently used (68, 69). Opioids are only recommended as rescue analgesics. Codeine is forbidden in many countries for children and tramadol in some countries too. Especially in the United States, strategies are searched to avoid opioids in consequence of the opioid epidemic (70). Optimal combination of paracetamol plus NSAID might decrease the probability to need opioids as rescue analgesics (71).

The analgesic effect of intraoperative and postoperative acupuncture as well as postoperative honey was investigated (72–74). Acupuncture is rarely provided as it requires specific training. The optimal acupuncture regime has not been defined, yet. More randomized controlled trials are needed to define the risk and combination of most effective drugs for postoperative pain relief after tonsillectomy.

**Recommendation:** An essential part of tonsillectomy is perioperative and postoperative pain management. Postoperative management should include a combination of paracetamol and NSAIDs as the first choice treatment unless contraindicated due to other reasons.

### Ongoing clinical trials and clinical registers

After decades without important trials, the NATTINA trial was the start of a series of studies that are still ongoing (Supplementary Table S3). The German TOTO trial is a multicenter, 1:1 two-arm, randomized non-blinded non-inferiority trial (14). The non-inferiority of tonsillotomy compared to tonsillectomy is investigated. Patients ≥3 years of age are randomly allocated to undergo either tonsillotomy or tonsillectomy as surgical treatment of recurrent acute tonsillitis. The primary outcome is the number of sore throat days experienced over the 24-month follow-up. The Finnish FINITE trial investigates the hypothesis that the recovery time from tonsil surgery can be reduced with intracapsular tonsillectomy (75). Adult patients suffering from recurrent or “chronic” tonsillitis are included into a randomized, controlled, three-arm clinical trial. It is designed to compare three different surgical techniques: extracapsular monopolar tonsillectomy, intracapsular microdebrider tonsillectomy, and intracapsular coblation tonsillectomy. The primary endpoint is the recovery time from postoperative pain. Another Finnish multicenter trial compared tonsillectomy, tonsillotomy, and watchful waiting in adult patients with recurrent or “chronic” tonsillitis (76). The primary outcome will be tonsillitis-specific quality of life at 6 months. These trials will give important answers on the efficacy of tonsil surgery in adults and especially if less than total tonsillectomy will be effective.

Concerning clinical registers, the Nordic countries present good examples. In Sweden, the National Tonsil Surgery Register (https://ton.registercentrum.se/) is used since 1997 (77). Such quality registers have also been established in Norway and Denmark since 2016 (78). With the aim to improve the care of patients needing tonsil surgery, these registers allow an ongoing investigation of indications, surgical methods, and complications. In addition, national healthcare data form an important source for quality control of tonsil surgery. For instance, using a national cohort of children from the US American Pediatric Health Information System allowed an analysis of 96,415 children undergoing tonsillectomy between 2016 and 2021 (58). Important variables associated with postoperative bleeding were revealed including a probability model for future quality initiatives.

**Recommendation:** The role of tonsillotomy for recurrent acute tonsillitis in children as well as in adults is unclear. The role of different partial tonsillotomy techniques and if children and adults have to be treated differently is anticipated through ongoing clinical trials. Beyond clinical trials, national quality registers and national healthcare data represent underutilized opportunities to improve care of patients with recurrent acute tonsillitis.

## Discussion

The data of actual clinical trials on a high evidence-based level allowed us to define clear recommendations for the treatment of patients with recurrent acute tonsillitis, most important for the indication of antibiotic treatment and for tonsillectomy. If the patient presents a history of ≤7 sore throats in the preceding year, <5 episodes in each of the preceding 2 years, or <3 episodes in each of the preceding 3 years, and the new episode has a high score (Centor, McIsaac, FeverPAIN), especially when presenting with a positive GAS POCT, antibiotic treatment can be considered. If the patient experiences more episodes, i.e., at least five to seven sore throat episodes within 1–2 years, bilateral tonsillectomy can be recommended as the patient will profit much more from surgery than from another conservative treatment.

Nevertheless, the literature research clearly showed that there are many areas of uncertainty with an urgent need for more objective criteria to define the severity of a recurrent acute tonsillitis. The number of tonsillitis episodes per year cannot be exactly established and, therefore, is a non-reliable inclusion criterion. Furthermore, no reliable preoperative biomarkers are available to predict the outcome of tonsillectomy (45, 79). Machine learning algorithms to classify the clinical optical appearance of the tonsil might suggest or add an alternative solution, but with the number of tonsillitis episodes to train such algorithms, their meaningfulness will be limited (80). Innovative tools allowing a non-invasive cellular and molecular imaging and severity classifying of the tonsils are warranted. This might be a way to classify the severity without removing the tonsils and could spare some unneeded surgeries (81, 82).

The worldwide decline of tonsillectomy is associated with more acute admissions for tonsillitis in some countries (83). There seems to be a correlation between social deprivation and both tonsillectomy and tonsillitis; hence, lower socioeconomic groups are more effected. The reasons for this appear to be multifactorial. We also might need criteria reflecting the burden of the disease on socioeconomic factors, i.e., beyond the current approach of counting of tonsillitis episodes (83).

Finally, there is an ongoing debate on the long-term effects of tonsillectomy, i.e., the removal of a secondary immune organ. Tonsillectomy does not seem to have a negative effect on the immune system, when humoral and cellular immunity is measured after surgery (84–86). During the COVID-19 pandemic, it was speculated that patients who underwent a tonsillectomy might have a higher risk for a more severe course of the SARS-CoV2 infections (87, 88). This has remained so far unproven (89). Several population-based studies have been performed to study the effects of tonsillectomy on later occurrences of other diseases. The risk of pneumonia after tonsillectomy in adults seems not to be increased (90), whereas the risk for later development of an autoimmune disease seems to be slightly increased (91, 92). The list of studies investigating the risk of cancer after prior tonsillectomy seems to be larger: there is a slightly increased risk for breast cancer (93, 94) and Hodgkin’s disease (95). Of interest is a (self-explanatory) lower risk of oropharyngeal cancer (96). However, the causal relation remains unclear. It might be that factors that led to recurrent acute tonsillitis are responsible for this relation and not the tonsillectomy itself (97, 98). Hence, due to several reasons, more long-term studies after tonsillectomy are needed. Register studies might be the best option to harvest long-term data after tonsil surgery.

## Conclusion

Recurrent acute tonsillitis worldwide has a high prevalence in children and adults. Each sore throat/tonsillitis episode should be classified by one of the three international scoring systems. In addition, a tonsillitis-specific quality of life score should be used. It is important both to classify the severity of the disease of each new episode and to further guide the need for an additional GAS POCT. These estimations are important requirements to decide for the optimal analgesic regime and, if the probability of a bacterial infection is high, to recommend for an antibiotic treatment. There is high evidence that tonsillectomy decreases the rate of sore throats in the severely affected cases with a high frequency of episodes. It still remains unclear whether or not partial tonsillectomy strategies are as effective, and this will be investigated in the ongoing clinical trials. In case of tonsillectomy, standardized perioperative and postoperative pain management is mandatory.
