# Supplementary material for: Treatment of recurrent acute tonsillitis—a systematic review and clinical practice recommendations
Source: Front Surg. 2023 Oct 10;10:1221932. doi: 10.3389/fsurg.2023.1221932 (PMC10597714; doi:10.3389/fsurg.2023.1221932)

Supplemental Material

Supplement Fig. 1

Treatment of Recurrent Acute Tonsillitis– a Systematic Review and Clinical Practice Recommendations

Orlando Guntinas-Lichius[1], Katharina Geißler[1], Antti A. Mäkitie[2], Ohad Ronen[3], Patrick J. Bradley[4], Alessandra Rinaldo[5], Robert P. Takes[6], Alfio Ferlito[7]

Supplement Fig. 1. Preferred reporting items for Systematic reviews and meta-analyses (PRISMA) flow diagram of the literature selection process.

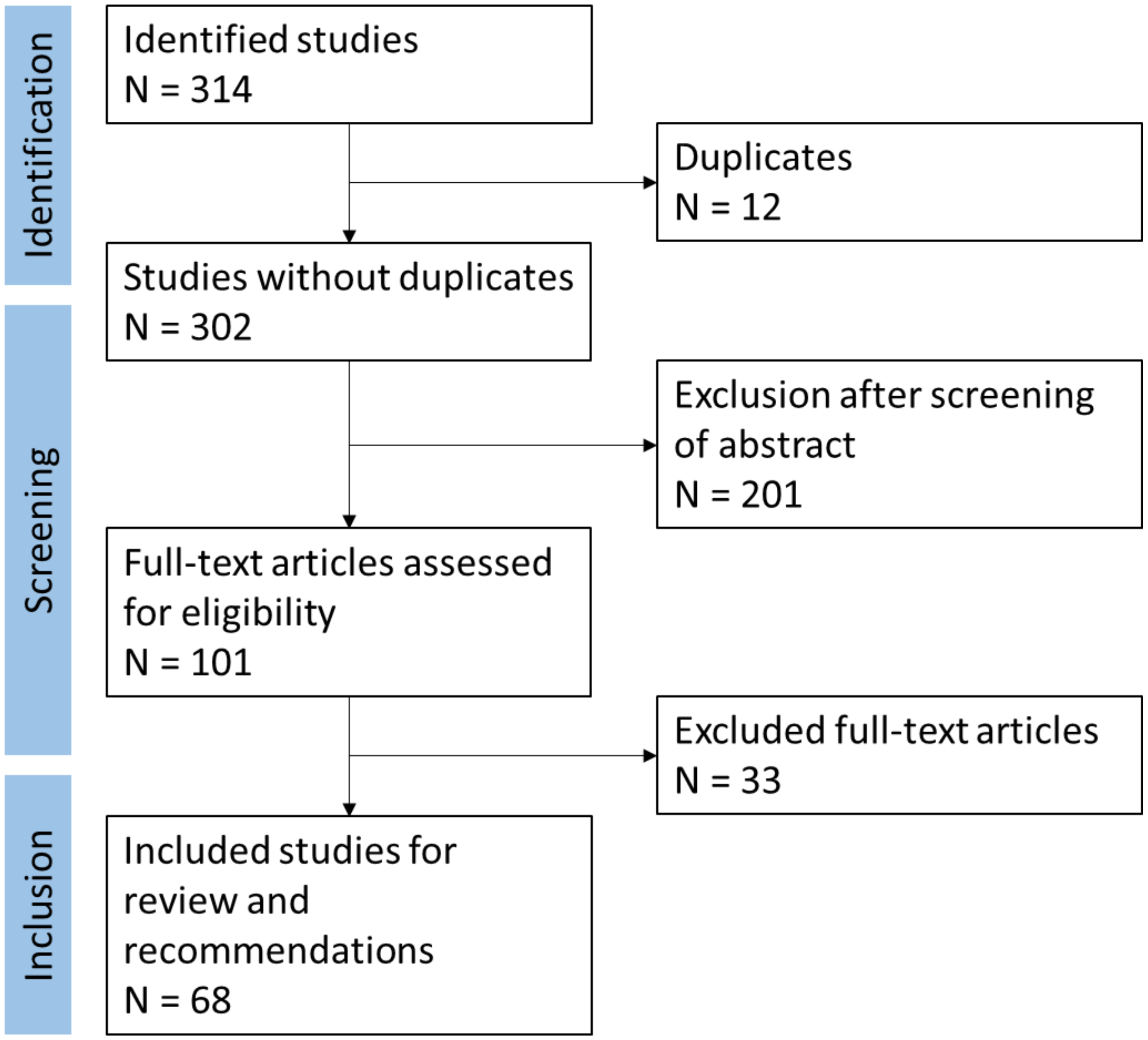

Supplement: Supplementary file 1 [file Datasheet1.pdf]
