# Supplementary material for: Treatment of recurrent acute tonsillitis—a systematic review and clinical practice recommendations
Source: Front Surg. 2023 Oct 10;10:1221932. doi: 10.3389/fsurg.2023.1221932 (PMC10597714; doi:10.3389/fsurg.2023.1221932)
Supplement: Supplementary file 2 [file Datasheet2.pdf]

Supplemental Material

Supplementary Tables S1, S2, S3

Treatment of Recurrent Acute Tonsillitis– a Systematic Review and Clinical Practice Recommendations

Orlando Guntinas-Lichius, Katharina Geißler, Antti A. Mäkitie, Ohad Ronen, Patrick J. Bradley, Alessandra Rinaldo, Robert P. Takes, Alfio Ferlito

The references in brackets are referring to the reference list in the main document.

The supplement material contains three additional references:

*. Materia E, Baglio G, Bellussi L, Marchisio P, Perletti L, Pallestrini E, et al. The clinical and organisational appropriateness of tonsillectomy and adenoidectomy-an Italian perspective. Int J Ped Otorhinolaryngol 2005;69(4):497-500. doi: 10.1016/j.ijporl.2004.11.016.

**. Lescanne E, Chiron B, Constant I, Couloigner V, Fauroux B, Hassani Y, et al. Pediatric tonsillectomy: clinical practice guidelines. Eur Ann Otorhinolaryngol Head Neck Dis 2012;129(5):264-71. doi: 10.1016/j.anorl.2012.03.003.

***. (SIGN) SIGN. Management of sore throat and indications for tonsillectomy. A national clinical guideline. Edinburgh: SIGN publication; 2010.

Supplementary Table S1

**Supplementary Table S1.** Important clinical guidelines for tonsil surgery in case of recurrent acute tonsillitis.		
**Society/authors; publication date**	**Guideline**	**Comment**
American Academy of Otolaryngology–Head and Neck Surgery Foundation (19); published 2019	Clinical Practice Guideline: Tonsillectomy in Children (Update)	Guideline only for children. This is a tonsillectomy guideline, i.e. also other indications than recurrent acute tonsillitis are considered. Indication for tonsillectomy for recurrent acute tonsillitis are based on Paradise criteria (see **Table 2**).
Italian National Programme for Clinical Guidelines (*); published 2005	Clinical and organisational appropriateness of tonsillectomy and adenoidectomy	For children and adults for several indications, not only for recurrent acute tonsillitis. Consensus process not described.
German Society of Oto-Rhino-Laryngology, Head and Neck Surgery (16); published 2015	Therapy of inflammatory diseases of the palatine tonsils - tonsillitis	For children and adults. Only available in German language. This is a guideline for treatment of acute tonsillitis and recurrent acute tonsillitis.
French Society of ENT, French Association for Ambulatory Surgery (AFCA), French Society for Anaesthesia, Intensive Care (**); published 2012	Pediatric tonsillectomy clinical practice guideline	Guideline only for children. This is a tonsillectomy guideline, i.e. also other indications than recurrent acute tonsillitis are considered. Tonsillar hypertrophy as indication is also addressed. Furthermore, this is the only guideline still using the term “chronic tonsillitis”. In distinction to recurrent acute tonsillitis, this term is used for inflammatory symptoms for ≥3 months recalcitrant medical treatment. We have doubts that such a disease exists.
Scottish Intercollegiate Guidelines Network (SIGN) (***); published 2010	Management of sore throat and indications for tonsillectomy - A national clinical guideline	Guideline for children and adults refers to sore throat episodes due to acute tonsillitis.

Supplementary Table S2

**Supplementary Table S2.** Paradise criteria and its modifications for indication of tonsil surgery in case of recurrent acute tonsillitis.					
**Parameters**	**Original Paradise criteria used in the American Academy of Otolaryngology–Head and Neck Surgery Foundation guideline (4)**	**German guideline: Therapy of inflammatory diseases of the palatine tonsils – tonsillitis (16)**	**Italian clinical and organisational guideline (*)**	**French pediatric tonsillectomy: Clinical practice guidelines (**)**	**Scottish Intercollegiate Guidelines Network Management of sore throat and indications for tonsillectomy guideline (***)**
Sore throat episodes	• ≥7 episodes of sore throat in the preceding year, OR<br>• ≥5 episodes in each of the preceding 2 years, OR<br>• ≥3 episodes in each of the preceding 3 years	• ≥6 episodes of acute tonsillitis in the preceding year, OR<br>• 3-5 episodes in the preceding year, if ≥6 episodes are reached within the following 6 months	• ≥5 bacterial episodes in the preceding year, AND<br>• disabling and prevent normal functioning, yet only after an additional observational period of at least 6 months	• ≥ 3 episodes of infection per year over a 3-year period OR<br>• 5 episodes over 2 years	• ≥7 more well documented, clinically significant, adequately treated sore throats in the preceding year OR<br>• ≥5 such episodes in each of the preceding 2 years OR<br>• ≥3 such episodes in each of the preceding 3 years.
Clinical symptoms	• Temperature >38.3° C (>101° F), OR<br>• Cervical lymphadenopathy (tender lymph nodes or >2 cm), OR<br>• Tonsillar exudate, OR<br>• Positive culture for group A beta-	• Tonsil size > Brodsky grade 1 (54) if a tonsillotomy is planned	• More flexibility in the presence of significant and persistent laterocervical lymphoadenopathy; episodes of peritonsillar abscess; OR febrile convulsions		• sore throats are due to acute tonsillitis<br>• the episodes of sore throat are disabling and prevent normal functioning

	hemolytic streptococcus				
Prior treatment	• Antibiotics had been administered in conventional dosage for proved or suspected streptococcal episodes				
Documentation requirements	• Each episode and its qualifying features had been substantiated by contemporaneous notation in a clinical record, OR<br>• If not fully documented, subsequent observance by the clinician of 2 episodes of throat infection with patterns of frequency and clinical features consistent with the initial history	• The episodes have been diagnosed by a physician and were treated with antibiotics			
Type of surgery	• Tonsillectomy	• Tonsillectomy OR tonsillotomy	• Tonsillectomy	• Tonsillectomy	• Tonsillectomy

Supplementary Table S3

Supplementary Table S3. Ongoing randomized controlled trials including patients with recurrent acute tonsillitis.							
**Authors**	**Age, years**	**Numbers planned**	**Eligibility**	**Intervention (I) Comparator ©**	**Primary outcome**	**Follow-up**	**Started/ completion planned**
Guntinas-Lichius et al. (14)	≥3	454	• ≥ 6 episodes of physician-diagnosed tonsillitis treated with antibiotics within the last 12 months OR<br>• 3–5 episodes, if (a) further episodes occur in the next 6 months and a total number of 6 episodes is reached, OR<br>• (b) there are additional patient-specific factors (special personal or professional concern, severe symptoms during the tonsillitis episodes, individual patient request) according to the German guideline for the treatment of tonsillitis (4).	I1: Tonsillotomy<br>I2: Tonsillectomy	number of sore throat days experienced over the 24-month follow-up	24 months	2020/2026
Laajala et al. (76)	≥18	285	• ≥3 episodes in 6 months OR<br>• ≥4 episodes in 12 months OR<br>• chronic tonsillitis, i.e. recurrent or chronic throat pain for ≥6 months, ≥1	I1: Tonsillotomy<br>I2: Tonsillectomy	Tonsillectomy Outcome Inventory-14 scores at 6 months	5-6 months	2020/2023

			symptom or sign must indicate that symptoms originate from the palatal tonsils:<br>• disturbing tonsil stones, halitosis, anterior cervical lymphadenitis, tonsillar exudates, abnormal tonsillar crypts. Symptomatic treatment has not been effective.				
Piitulainen et al. (75)	16–65	165	• ≥3 episodes of tonsillitis in the last 12 months, OR<br>• chronic tonsillitis, i.e prolonged tonsil-derived throat pain and ≥1 symptom: enlarged tonsils, tonsillar exudates, halitosis,<br>• tonsillar stones, enlarged and tender submandibular lymph nodes	I1: extracapsular monopolar tonsillectomy<br>I2: intracapsular microdebrider tonsillectomy<br>I3: intracapsular coblation tonsillectomy	Recovery time from surgery	60 months	2019/2027
